# Is it possible to prevent recurrent vulvovaginitis? The role of *Lactobacillus plantarum* I1001 (CECT7504)

**DOI:** 10.1007/s10096-016-2715-8

**Published:** 2016-07-09

**Authors:** S. Palacios, J. Espadaler, J. M. Fernández-Moya, C. Prieto, N. Salas

**Affiliations:** 1 Instituto Palacios de Salud y Medicina de la Mujer, Antonio Acuña 9CP, 28009 Madrid, Spain; 2 Autonomous University of Barcelona, AB-Biotics S.A. Eureka building, 08193 Bellaterra, Barcelona Spain; 3 Instituto de Medicina EGR, Camino de la Zarzuela, 19, 28023 Aravaca, Madrid Spain; 4 Gynea Laboratorios by Kern Pharma, Pol. Ind. Colom II, C/Venus, 72, 08228 Terrasa, Barcelona Spain

## Abstract

The purpose of this study was to prospectively evaluate the impact of the use of *L. plantarum* I1001 applied vaginally on Vulvovaginal Candidiasis (VVC) time-until-recurrence after treatment with single-dose vaginal clotrimazole. This was a clinical open-label, prospective study of two non-randomized parallel cohorts with symptomatic acute VVC: (1) 33 sexually active women 18–50 years old, prescribed a standard single-dose 500 mg vaginal tablet of clotrimazole followed by vaginal tablets with *L. plantarum* I1001 as adjuvant therapy, and (2) 22 women of similar characteristics but prescribed single-dose clotrimazole only. Use of the probiotic and factors that might influence recurrence risk (age, recurrent VVC within previous year, antibiotic prior to study enrolment, diaphragm or IUD contraception, among others) were included in a multivariate Cox regression model to adjust for potential between-cohort differences. Probiotic use was associated with a three-fold reduction in the adjusted risk of recurrence (HR [95 %CI]: 0.30 [0.10–0.91]; P = 0.033). Adjusted free-survival recurrence was 72.83 % and 34.88 % for the probiotic and control groups, respectively. A higher cumulative recurrence was also observed in cases with use of antibiotics prior to enrolment (HR [95 %CI]: 10.46 [2.18–50.12]; P = 0.003). Similar findings were found at six months after azole treatment in women with RVVC. Overall, good compliance with the probiotic was reported for 91.3 % of women. The study suggests that follow-up therapy with vaginal tablets with *L. plantarum* I1001 could increase the effectiveness of single-dose 500 mg clotrimazole at preventing recurrence of VVC, an effect that was also observed in women with recurrent vulvovaginal candidiasis (RVVC) after six months of azole treatment.

**Electronic supplementary material:**

The online version of this article (doi:10.1007/s10096-016-2715-8) contains supplementary material, which is available to authorized users.

## Introduction

Vulvovaginal candidiasis (VVC) is one of the most frequent infections of the female genital tract and is mainly associated with *Candida albicans* (followed by *Candida glabrata)* [1]. *Candida* species are normally found in the lower genital tract of 10–20 % of healthy women of childbearing age [2]. Evolution from colonization to symptomatic infection involves different host factors such as susceptibility and inflammatory responses and/or the imbalance of vaginal microbiota [3].

Available literature on VVC suggests that 75 % of women will show a VVC episode [4, 5] through their lifetime, and that 5–10 % of all women will experience recurrent vulvovaginal candidiasis (RVVC), i.e ≥4 episodes/year [6].

Short-term and single-dose vaginal antifungals are successful in 80–90 % of acute uncomplicated cases [7, 8]. However, azole resistance rates are above 15 % in women with RVVC [9, 10]. Antifungal resistance, infection recurrence and side effects of pharmacological treatments are key issues for patients and their physicians seeking alternative interventions for VVC.

Probiotics are defined as “live microorganisms, which when administered in adequate amounts, confer a health benefit on the host” [11]. Probiotic properties are strain-specific so their positive health effects cannot be extrapolated to other strains of the same species or genus [12]. Lactobacilli are the predominant bacteria in a healthy vaginal ecosystem, and have been proposed for the treatment and prevention of genitourinary infections including candidiasis [4, 6]. Local use of these microorganisms results in immunomodulation responses and the restoration of vaginal microbiota, interfering with the colonization and growth of potential pathogens such as Candida [13]. A Lactobacilli-dominated vaginal microbiota produces significant levels of lactic acid with strong microbicidal properties [14].

Evidence of synergy between adjuvant local Lactobacilli and azole treatment for VVC has been reported [9, 15]. Nevertheless, evidence of the benefit of adding probiotics to the standard azole on the risk of symptomatic recurrence is lacking.

Recently, a pilot study for the evaluation of colonization and tolerability of a vaginal tablet of *L. plantarum* strain I1001 (deposit code CECT7504) in healthy women, reported that use of this formulation, three times a week on alternate days, achieves an adequate vaginal lactobacilli concentration [16]. These positive results led us to prospectively evaluate the impact of the use of *L. plantarum* I1001, applied vaginally, on VVC recurrence after a single-dose vaginal clotrimazole.

## Patients and methods

A clinical open-label, prospective study of two non-randomized parallel cohorts was conducted in the out-patient gynaecological departments of the Instituto Palacios de Salud y Medicina de la Mujer and the Instituto de Medicina EGR (Madrid) from June 2013 until February 2015.

The first cohort (n = 33) corresponded to sexually active women between 18 and 50 years old with symptomatic acute VVC, who were prescribed with a standard single-dose 500 mg vaginal tablet of clotrimazole, followed by the *L. plantarum* I1001 vaginal probiotic tablet (Melagyn^®^ Probiotico Vaginal, Gynea by Kern Pharma, Spain; each tablet contains *L. plantarum* I1001 at minimum 1 × 10^8^ colony forming units) as adjuvant therapy (one tablet, three times a week on alternate days, for two consecutive months, excluding days with menstruation). The second cohort (n = 22) compromised women of similar characteristics but prescribed the single-dose clotrimazole treatment only.

Exclusion criteria (both cohorts) were pregnancy/breast feeding, delivery or abortion within the previous trimester, signs of other vaginal infections, abnormal genital bleeding within the previous semester, or any concomitant medication during follow-up that might significantly influence the evaluation and/or study results (including beta-lactams, clindamycin and tetracycline). Informed written consent was obtained from all participants prior to enrolment. All the study materials were approved by the Ethical Committee of the Hospital Universitario de la Princesa (Madrid, Spain).

### Vaginal probiotic and dose administration

*L. plantarum* I1001 (CECT7504) is a selected, patented strain which has been tested *in vitro*, showing good adherence to vaginal epithelial cells (VEC), high acidification of simulated vaginal media, high tolerance to antimicrobial factors of inflamed vaginal fluid, and intrinsically resistant to high concentrations of some typical antibiotics (ATB) and antifungals used for vaginal affections. This *L. plantarum* I1001 formulation has been previously tested in healthy women during a pilot open label clinical trial at Hospital Vall d’Hebron (Barcelona, Spain) [16], showing successful colonization of the vagina for at least 48 hours, therefore, allowing its application on alternate days.

### Data collection and follow-up

The primary outcome was the recurrence-free survival of signs/symptoms of VVC, as collected in the follow-up visits at two and three months after the azole treatment, within the possibility of a six-month visit for women with history of RVVC.

Specific risk factors (age, diabetes mellitus, RVVC within the last year, ATB prior to enrolment, diaphragm/intrauterine device or oral contraception, immunosuppression and history of other non-candida vulvovaginitis [VV]) were recorded at baseline. Self-reported presence of pruritus, vulvar soreness/irritation and burning vulvar pain, and signs of vaginal discharge, vulvar erythema and edema, and malodorousness, were assessed using a semi-quantitative scale (0 = absent, 1 = light, 2 = moderate and 3 = severe). In the *L. plantarum* I1001 cohort, physicians rated the effectiveness and tolerability of the product, using a semi-quantitative scale (0 = inadequate, 1 = fair, 2 = good, 3 = very good), while women also reported their opinion on the product and evaluated its tolerability and their satisfaction using the Spanish version of the Treatment Satisfaction Questionnaire for Medication (TSQM version 1.4) [17].

### Statistical analysis

Baseline characteristics (including known risk factors for VVC) of both cohorts were compared with Fisher’s exact test (categorical variables) and Mann-Whitney’s U test (quantitative variables). Values were summarized and presented as percentages or mean ± standard deviation (SD). For the main clinical outcome (VVC recurrence-free survival), use of the vaginal probiotic and additional factors that might influence the risk of recurrence (see above) were included in a multivariate Cox (proportional-hazards) regression model; these results are presented as Hazard ratios (HR) and their 95 % confidence intervals (95 %CI). Drop-outs were censored at the time of last follow-up. In an attempt to assess the change in recurrence within cohorts, we compared the average number of VVC episodes per trimester within 12 months prior to enrolment including the baseline episode of women with three-months follow-up (obtained from clinical records) to those observed during the follow-up at three months, by using the non-parametric Wilcoxon signed-rank test. Only subjects with complete clinical record for the past 12 months and complete follow-up at three months were considered for this later analysis. Results on the TSQM are presented as median and interquartile range (IQR), because of their non-normality. No imputation was performed for missing values, and significance was set at a level of 5 %, two-tailed. All statistical tests were performed with SPSS Statistics for Windows v22.0 (IBM Corp. 2013. Armonk, NY).

## Results

### Baseline data

**Fig. 1:**
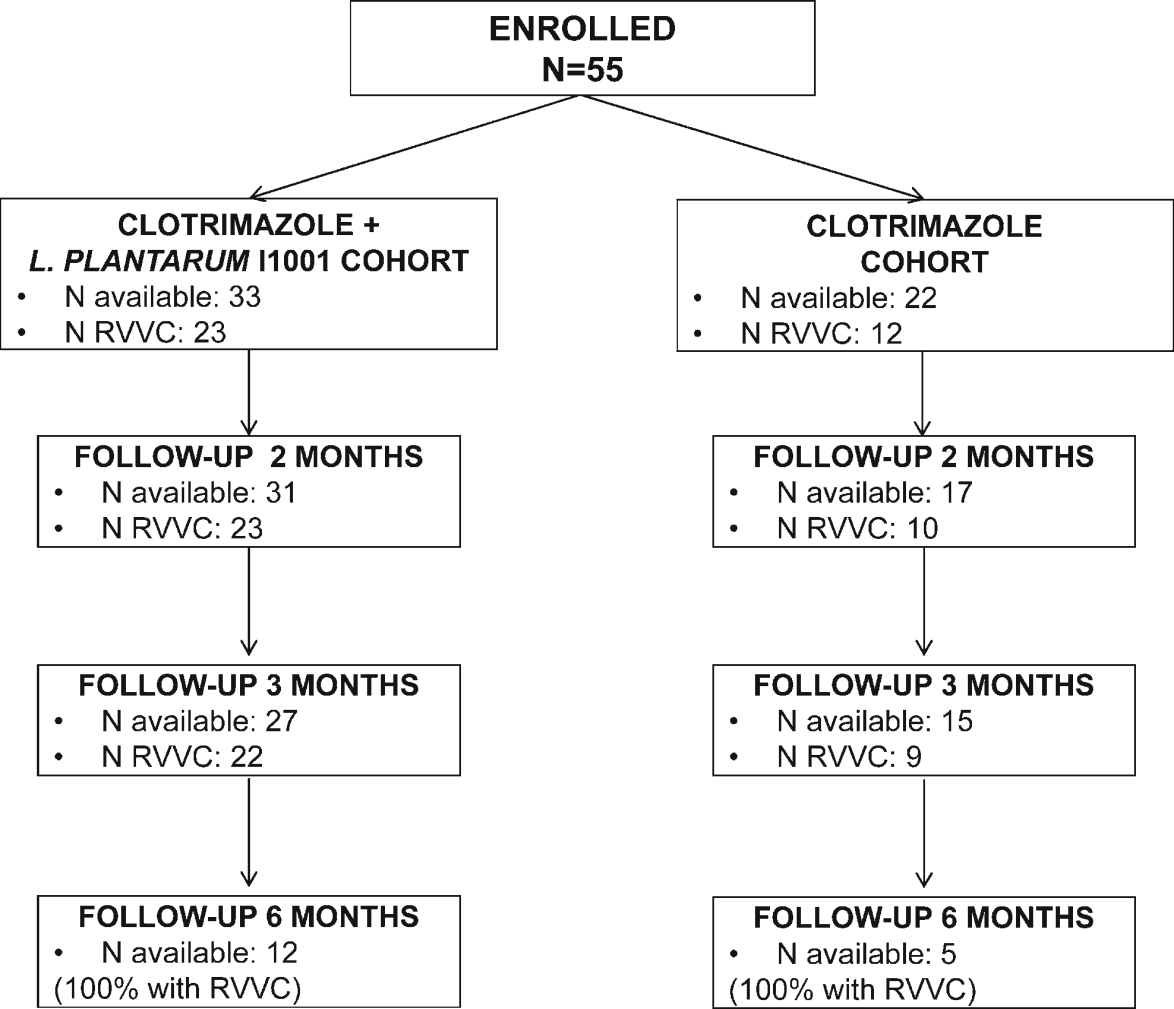
Patients flow-chart across study visits

A general description of the patients and current VVC episode is presented in Table 1. Flow of patients throughout the study is presented in Fig. 1. Drop-out rate was slightly higher in the clotrimazole-only cohort (18.5 % vs. 5.7 % at 2 months, and 24.1 % vs. 15.4 % at 3 months), although the difference did not reach statistical significance.

**Table 1:** Demographic, reproductive and gynaecological characteristics

Characteristic	Total sample	*Clotrimazole + L. plantarum* I1001	*Clotrimazole*	P-value
Total cases	55	33	22	
Age (years)	33.36 ± 8.61	33.58 ± 8.23	33.05 ± 9.34	0.830
Marital status				
Single	27 (49.1 %)	16 (48.5 %)	11 (50.0 %)	0.190
Divorced	2 (3.6 %)	0 (0.0 %)	2 (9.1 %)	
Married/living together	26 (47.3 %)	17 (51.5 %)	9 (40.9 %)	
Gynaecological history and reproductive state				
Child bearing age	51 (92.7 %)	32 (97.0 %)	19 (86.4 %)	0.290
Perimenopause	4 (7.3 %)	1 (3.0 %)	3 (13.6 %)	
Children (yes)	19 (34.5 %)	10 (30.3 %)	9 (40.9 %)	0.564
Relevant medical and/or surgical history	11 (20.4 %)	9 (28.1 %)	2 (9.1 %)	0.167
Risk factors for VV				
Antibiotics prior to study enrolment^a^	6 (11.5 %)	4 (12.9 %)	2 (9.5 %)	1.000
Diaphragm or IUD contraception^a^	3 (5.8 %)	2 (6.5 %)	1 (4.8 %)	1.000
Oral contraception^a^	11 (21.2 %)	6 (19.4 %)	5 (23.8 %)	0.739
Diabetes mellitus^a^	0 (0 %)	-	-	-
Immunosuppression^a^	1 (1.9 %)	1 (3.2 %)	0 (0.0 %)	1.000
Others^a^	4 (7.7 %)	2 (6.5 %)	2 (9.5 %)	1.000
Vulvovaginitis history				
Other non-candida VV	3 (5.6 %)	0 (0.0 %)	3 (13.6 %)	0.059
Any previous VVC	50 (90.9 %)	29 (87.9 %)	21 (95.5 %)	0.638
Age at first VVC episode^b^	25.15 ± 7.73	25.14 ± 8.07	25.17 ± 7.37	0.742
Total VVC episodes ever^b^	9.54 ± 7.96	9.66 ± 6.04	9.38 ± 10.20	0.242
RVVC ever^a^	35 (67.3 %)	23 (69.7 %)	12 (63.2 %)	0.761
Total VVC in the last 12 months^c^	4.22 ± 2.40	4.55 ± 2.58	3.53 ± 1.88	0.142
Any previous VVC in the last 12 months^a^	33 (63.5 %)	23 (69.7 %)	10 (52.6 %)	0.246
Last VVC episode				
Months since last VVC^d^	4.06 ± 3.73	3.72 ± 3.49	4.62 ± 4.13	0.413
Symptomatic days during the last VVC^e^	4.36 ± 2.55	4.31 ± 2.71	4.43 ± 2.36	0.719
VVC signs and symptoms				
Pruritus		25 (75.8 %)	19 (86.4 %)	0.495
Burning pain/soreness		25 (75.8 %)	20 (90.9 %)	0.284
Vulvar erythema		29 (87.9 %)	21 (95.5 %)	0.638
Vaginal discharge		28 (84.8 %)	20 (90.9 %)	0.689
Vulvar pain/dyspareunia		17 (51.5 %)	12 (54.5 %)	1.000
Edema		27 (81.8 %)	19 (86.4 %)	0.727
Malodourness		11 (33.3 %)	7 (31.8 %)	1.000

*VV* vulvovaginitis, *VVC* vulvovaginal candidiasis, *IUD* intrauterine device, *RVVC* recurrent vulvovaginal candidiasis

Values are presented as percentages or mean ± standard deviation (SD). Percentages are calculated based on the total number of patients available for each item. Comparison among the two intervention groups (P value) was performed with Fisher's test (categorical variables) or Mann Whitney test (quantitative variables)

^a^Data available for 52 patients^b^Data available for 47 patients ^c^Data available for 46 patients ^d^ Data available for 45 patients ^e^Data available for 50 patients

Among the 55 women from both cohorts, 50 (91 %) had a history of previous VVC anytime during their lifespan (Table 1), from which 33 (63.5 %) met criteria for VVC within the 12 months prior to enrolment. The number of VVC episodes during the previous 12 months was higher in the *L. plantarum* I1001 cohort (4.55 ± 2.58 vs. 3.53 ± 1.88), although this difference did not reach statistical significance. Conversely, previous non-Candida VVs were reported exclusively in the antifungal-only cohort (n = 3, P = 0.059, statistical trend).

Current VVC was generally characterized by the specialist with vulvar erythema, vaginal discharge, edema and burning/soreness, without differences between cohorts regarding the number of patients with each symptom.

### Patients’ and gynaecologists’ evaluation of the vaginal probiotic

Overall, patients treated with the vaginal probiotic reported a compliance of 91.3 %. The TSQM (n = 29) results showed that patients reported high ratings of efficacy, lack of side effects, convenience and overall satisfaction (Table 2). Only one patient reported a side effect (edema and erythema) of moderate intensity, and rated it as “somewhat affecting her satisfaction with the treatment” in the TSQM questionnaire (i.e. a score of 3 in a Likert 1–5 scale). Moreover, gynecologists considered efficacy of the test product in their patients (n = 31) as “good”/“very good” in 87 % of cases, and tolerability as “good”/“very good” in 100 % of them.

**Table 2:** Patients satisfaction with *L. plantarum* I1001 vaginal tablets (TSQM questionnaire)

Parameter	Total number of patients	Median	IQR
Efficacy	29	83.3	66.7–83.3
Side effects (lack of)	29	100	100–100
Convenience	29	83.3	66.7–83.3
Overall satisfaction	29	76.4	52.8–76.4

*IQR* interquartile range

### Time until symptomatic recurrence

Overall, 10/31 (32.2 %) of women in the probiotic group and 10/17 (58.8 %) of women in the control group had experienced one or more symptomatic recurrences during the 3–month follow-up (P = 0.125).

**Fig. 2:**
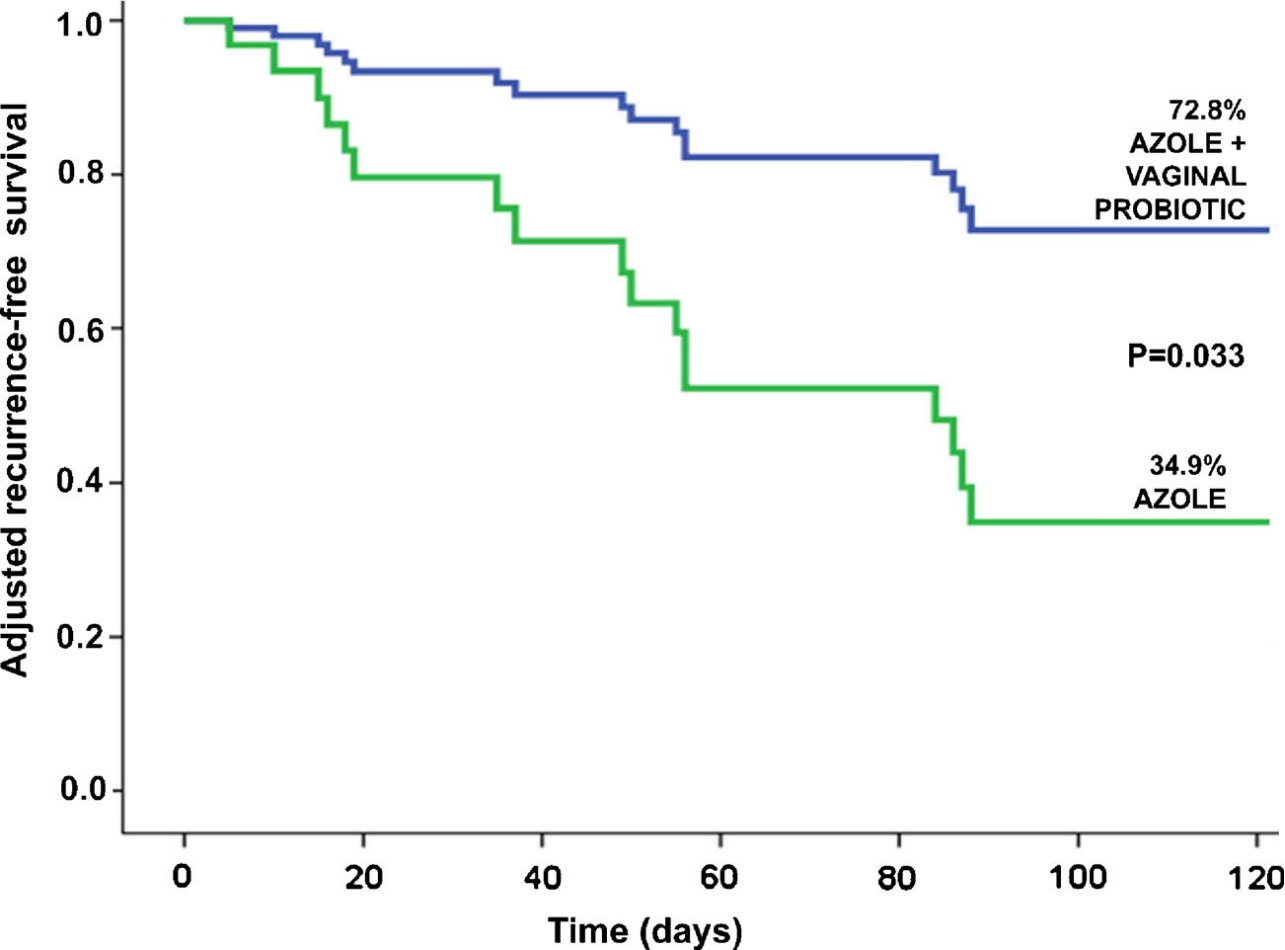
Recurrence-free survival curve 3 months after single-dose clotrimazole treatment. Comparison of the overall recurrence-free survival between patients treated with clotrimazole 500 mg single dose-only vs. clotrimazole + *L. plantarum* I1001. The difference on adjusted survival between the two cohorts is significant, with a higher recurrence-free survival in patients receiving adjuvant vaginal probiotic (HR [0.30 [95 %CI: 0.10–0.91]; P = 0.033, multivariate Cox regression model)

**Fig. 3:**
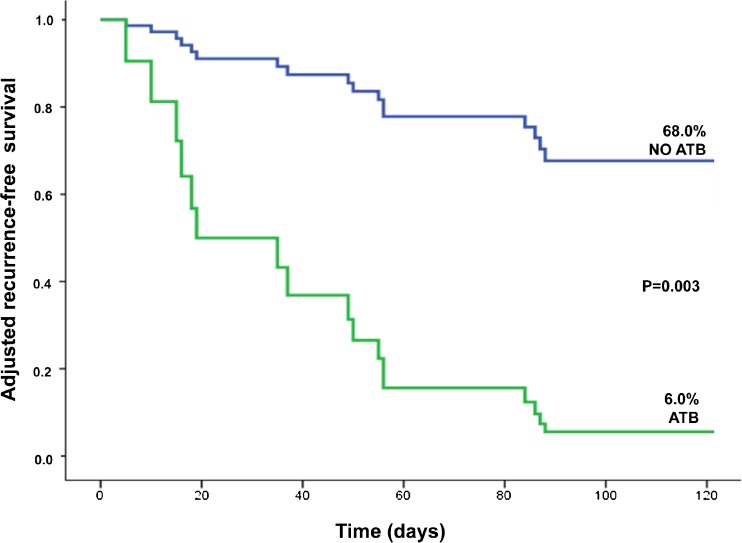
Recurrence-free survival curve 3 months after single-dose clotrimazole treatment. Patients with history of antibiotic treatment (ATB) prior to enrolment. Comparison of the overall recurrence-free survival depending on the use of antibiotic treatment prior to enrolment. The difference on adjusted survival between the two cohorts achieves significance with a markedly lower recurrence-free survival in patients with such risk factor (HR: 10.46 [95 %CI: 2.18–50.12]; P = 0.003, multivariate Cox regression model)

The Cox regression model on the combined cohorts (Table 3) showed that adjusted HR for developing a new VVC within three months of the single-dose clotrimazole, depending on probiotic use, was 0.30 (95 %CI: 0.10–0.91) and that this effect was statistically significant (P = 0.033, Fig. 2). Among other risk factors, ATB prior to enrolment also showed a significant effect, with an adjusted HR of 10.46 (95 %CI: 2.18–50.12); (P = 0.003, Fig. 3). Thus, comparison of the recurrence-free survival shows a clear increase in adjusted survival at 3 months (73 % vs. 35 %, i.e. a +108.6 % increase) in women receiving the probiotic, while the ‘previous ATB’ factor shows marked effect in the opposite direction (6 % vs. 68 %).

**Table 3:** Cox proportional-hazards regression model for recurrence of VVC at 3 months

Variable	Hazard ratio	95 % CI		P-value
		Lower	Upper	
Age	0.96	0.90	1.03	0.228
Any previous VVC within the last 12 months	1.69	0.49	5.79	0.406
Antibiotics prior to enrolment	10.46	2.18	50.12	0.003
Diaphragm or IUD contraception	1.55	0.30	7.91	0.600
Immunosuppression	0.42	0.03	5.15	0.493
Oral contraception	0.97	0.22	4.32	0.965
History of other non-candida VV	0.34	0.03	3.52	0.365
Use of *L. plantarum* I1001	0.30	0.10	0.91	0.033

*VVC* vulvovaginal candidiasis, *RVVC* recurrent VVC, *VV* vulvovaginitis, *IUD* intrauterine device, *CI* confidence intervals

Analysis of the subgroup with history of RVVC (n = 23 of the clotrimazole + probiotic cohort, and n = 12 from the clotrimazole cohort) at 6-months of follow-up, showed a similar difference in adjusted survival among treatment cohorts (63 % vs. 22 %, Supplementary material). The adjusted HR for symptomatic recurrence depending on probiotic use was 0.30 (95 %CI: 0.10–0.89; P = 0.03), likewise for the effect of ‘previous ATB’ (Supplementary material).

### Differences in the number of episodes

In the *L. plantarum* I1001 cohort the average number of VVC episodes per trimester during the 3-month follow-up was 0.45 ± 0.72, down from 1.14 ± 0.65 episodes/trimester during the 12 months prior to enrolment, thus representing a reduction of 59 % (P = 0.001). Conversely, such difference was not observed in the clotrimazole-only cohort (0.67 ± 0.62 vs. 0.88 ± 0.47 episodes/trimester; P > 0.1).

## Discussion

In the present study, follow-up therapy with vaginal tablets with *L. plantarum* I1001 after a single-dose 500 mg clotrimazole was found to significantly reduce the risk of symptomatic recurrence within 3 months after an acute VVC, with similar findings in the subset of patients with RVVC within 6 months of follow-up.

Although most baseline characteristics were comparable, patient distribution among cohorts was not randomized and resulted in a larger number of the VVC episodes of women with any previous VVC in the last 12 months and a different history of non-Candida VV in the probiotic cohort. Taking into account the lack of randomization and those differences between cohorts, we considered appropriate to use a multivariate survival test to study the primary outcome (the recurrence-free survival). After adjustment for potential confounding factors by Cox multivariate analysis, the effect of adjuvant probiotic was significant in the full study population (at 3-months) and in women with RVVC within a 6-month follow-up. Nevertheless, we were surprised to observe that the hazard ratio was virtually the same in the full population at 3 months and in the subpopulation with RVVC at 6 months. However, this lack of difference may be due to the small study sample. In future studies, it would be interesting to reproduce the same analysis using a larger sample in order to clear up whether there are any differences between the evaluation at 3- and 6-months of follow-up regarding the use of probiotics. Also, the difference in the fraction of women experiencing at least one symptomatic recurrence between the two study cohorts (32.2 % vs 58.8 %) did not reach statistical significance. However, this analysis at study endpoint does not take into account the baseline differences between the two cohorts, nor captures the time-to-event effect as the Cox analysis does. The latter may be of special relevance in the study population, as it was particularly prone to recurrence (67.3 % had a history of RVVC at any time of their lives and 63.5 % reported at least one previous VVC episode within the 12 months prior to enrolment).

Variability among candidiasis and the selection of treatment are issues that have aroused considerable discussion. Recent reports suggest the possibility of two types of VVC: (a) the typical, characterized by dense vaginal discharge with occasional recurrences, and (b) the cyclic-recurrent, with hypersensitive reactions even in presence of small quantities of Candida [4]. In both cases, a strong vaginal ecosystem is desirable to fend off Candida strains and reduce the risk of VVC recurrence. Several authors suggest that maintenance therapy needs to be given frequently enough to prevent vaginal regrowth or recolonization, and transformation to a symptomatic state in cases with “host intolerance”; however, once the azole is suspended, the risk of a new episode is particularly high [8].

Witt et al. [18] found no advantages in treating RVVC with monthly 200 mg itraconazole, despite the adjuvant use of local *L. gasseri* lyophilisates for 6 days. Thus, in our case, the probiotic intervention was maintained over 2 months to ensure proper concentrations of lactobacilli.

Probiotics are being used in the gynaecological field as an alternative intervention for VVC and its prevention after ATB therapy, and so for bacterial vaginosis. Evidence suggests that the coaggregation of lactobacilli prevents Candida from binding to VEC [19], therefore improving the efficacy of antifungals [20, 21]. A retrospective comparative study by De Seta et al. [21] showed a better subjective resolution of typical VVC symptoms after treatment with clotrimazole 2 % vaginal cream for 3 days followed by vaginal application of *L. plantarum* P17630 daily for 6 days, plus a weekly application for 4 weeks, all compared with azole cream alone. However, they excluded patients with known risk factors of VVC (RVVC, prior ATB, etc.). In our study, on the contrary, no exclusion of them was considered since these women are more likely to require supplementary therapies to standard treatments.

Despite the similarities between the published evidence and our results, it should be stressed that most reports have assessed multidose azole treatment [20, 21]; hence, the effects of the *L. plantarum* I1001 strain may be of particular interest since use of these adjuvant vaginal tablets was associated with a significant 3-fold reduction in the HR of recurrence, during a 3-month follow-up. To our knowledge, this is the first study to report a significant effect of a probiotic in the risk of symptoms recurrence in women with high prevalence of RVVC. It is noteworthy that ATB prior to enrolment significantly increased the VVC recurrence risk, consistent with previous reports such as those from Pirotta et al. [22] and Spinillo et al. [23], who also concluded that this factor could entail even a first-episode of VVC. Their findings and those presented herein enhance the role of vaginal probiotics as a preventive strategy (complementing pharmacological treatment), and a primary intervention for reducing the risk of vaginal infections. Since ‘Prior ATB’ *per se* seems to significantly increase probabilities of VVC and of recurrences, *L. plantarum* I1001 could be of relevance as a resource for women needing to replenish their vaginal microbiota after taking ATB.

It has to be noted that, in our study, VVC relapse was considered on the basis of any self-reported sign/symptom suggesting a new episode. Despite the subjective nature of these criteria, it could probably represent a more realistic and practical criteria in daily practice, considering that the pure presence of symptoms can entail a medical consultation and/or use of over-the-counter medication. Beyond the clinical impact of the probiotic, high rates of satisfaction, tolerability and effectiveness were reported by both patients and gynaecologists.

Our study has certain limitations that must be pointed out, such as the previously commented lack of randomization and the open-label nature of the trial, thus a placebo effect cannot be ruled out. However, it is worth pointing out that the high number of women fulfilling RVVC criteria suggests they may be well aware of the symptoms of a true VVC episode. And finally, the sample size available was limited, and drop-outs during the study follow-up were not uncommon. Thus, additional studies should be conducted to evaluate the effectiveness of *L. plantarum* I1001, using double blind randomized designs and larger sample sizes. Also, it would be interesting to evaluate the efficacy of *L. plantarum* I1001 as add-on or follow-up therapy of other antifungal regimes, as well as for preventing the recurrence of bacterial vaginosis.

## Electronic supplementary material

Below is the link to the electronic supplementary material.

Table S1 Cox proportional-hazards regression model for recurrence of VVC at 6 months

Table S2 Average number of VVC episodes per trimester within previous 12 months compared withthose observed during follow-up

Figure S1 Symptomatic recurrence at 3 months according to use of vaginal probiotic (*A*) and antibiotic treatment prior to enrolment (*B*)

Figure S2 Event-free survival curve at 6 months after single-dose azole treatment, depending on probiotic use. Comparison of overall recurrence-free survival between patients treated with clotrimazole 500 mg single dose vs. clotrimazole + *L. plantarum* I1001. The difference on adjusted survival between the two cohorts achieves significance with a higher recurrence-free survival in cases with adjuvant vaginal probiotic (HR: 0.30 [95 %CI: 0.10–0.89]; P = 0.03; multivariate Cox regression model)

Figure S3 Event-free survival curve – 6 months after single-dose azole treatment, depending on history ATB prior to enrolment. Comparison of overall recurrence-free survival regarding history of antibiotic treatment prior to enrolment. The difference on adjusted survival between the two cohorts achieves significance with a markedly higher recurrence-free survival rate in cases without such risk factor (HR: 7.87 [95 %CI: 1.81-34.26]; P = 0.006, multivariate Cox regression model)
