# Supplementary material for: Is it possible to prevent recurrent vulvovaginitis? The role of Lactobacillus plantarum I1001 (CECT7504)
Source: Eur J Clin Microbiol Infect Dis. 2016 Jul 9;35(10):1701–8. doi: 10.1007/s10096-016-2715-8 (PMC5035666; doi:10.1007/s10096-016-2715-8)
Supplement: Supplementary file 1 — Cox proportional-hazards regression model for recurrence of VVC at 6 months [file 10096_2016_2715_MOESM1_ESM.docx]

**SUPPLEMENTARY MATERIAL**

| **Table S1. Cox proportional-hazards regression model for recurrence of VVC at 6 months** | | | | |
| --- | --- | --- | --- | --- |
|  | OR | 95% CI | | P |
|  |  | Lower | Upper |  |
| Age | 0.97 | 0.91 | 1.03 | 0.318 |
| VVC Recurrence within the last 12 months | 1.68 | 0.48 | 5.85 | 0.414 |
| Previous antibiotics | 11.08 | 2.31 | 53.20 | 0.003 |
| Diaphragm or IUD contraception | 1.57 | 0.32 | 7.84 | 0.580 |
| Immunosuppression | 0.45 | 0.04 | 5.57 | 0.535 |
| Oral contraception | 1.25 | 0.29 | 5.34 | 0.768 |
| History of other non-candida VV | 0.55 | 0.08 | 3.88 | 0.551 |
| Use of *L. plantarum* I1001 | 0.30 | 0.10 | 0.89 | 0.030 |

VVC: Vulvovaginal Candidiasis; IUD: Intrauterine Device; VV: Vulvovaginitis; CI: Confidence Intervals.
