# Supplementary material for: Is it possible to prevent recurrent vulvovaginitis? The role of Lactobacillus plantarum I1001 (CECT7504)
Source: Eur J Clin Microbiol Infect Dis. 2016 Jul 9;35(10):1701–8. doi: 10.1007/s10096-016-2715-8 (PMC5035666; doi:10.1007/s10096-016-2715-8)
Supplement: Supplementary file 2 — Average number of VVC episodes per trimester within previous 12 months compared withthose observed during follow-up [file 10096_2016_2715_MOESM2_ESM.docx]

| **Table S2. Average number of VVC episodes per trimester within previous 12 months compared with those observed during follow-up.** | | | | | | |
| --- | --- | --- | --- | --- | --- | --- |
|  | *Clotrimazole +*  *L. plantarum* I1001 | | | *Clotrimazole* | | |
|  | n | Mean ± SD | P | n | Mean ± SD | P |
| Previous 12 months | 31 | 1.14 ± 0.65 | 0.001 | 15 | 0.88 ± 0.47 | 0.260 |
| During follow-up (3 months) |  | 0.45 ± 0.72 |  |  | 0.67 ± 0.62 |  |

VVC: Vulvovaginal Candidosis ; P value Wilcoxon
