# Supplementary figures and images for: Is it possible to prevent recurrent vulvovaginitis? The role of Lactobacillus plantarum I1001 (CECT7504)
Source: Eur J Clin Microbiol Infect Dis. 2016 Jul 9;35(10):1701–8. doi: 10.1007/s10096-016-2715-8 (PMC5035666; doi:10.1007/s10096-016-2715-8)

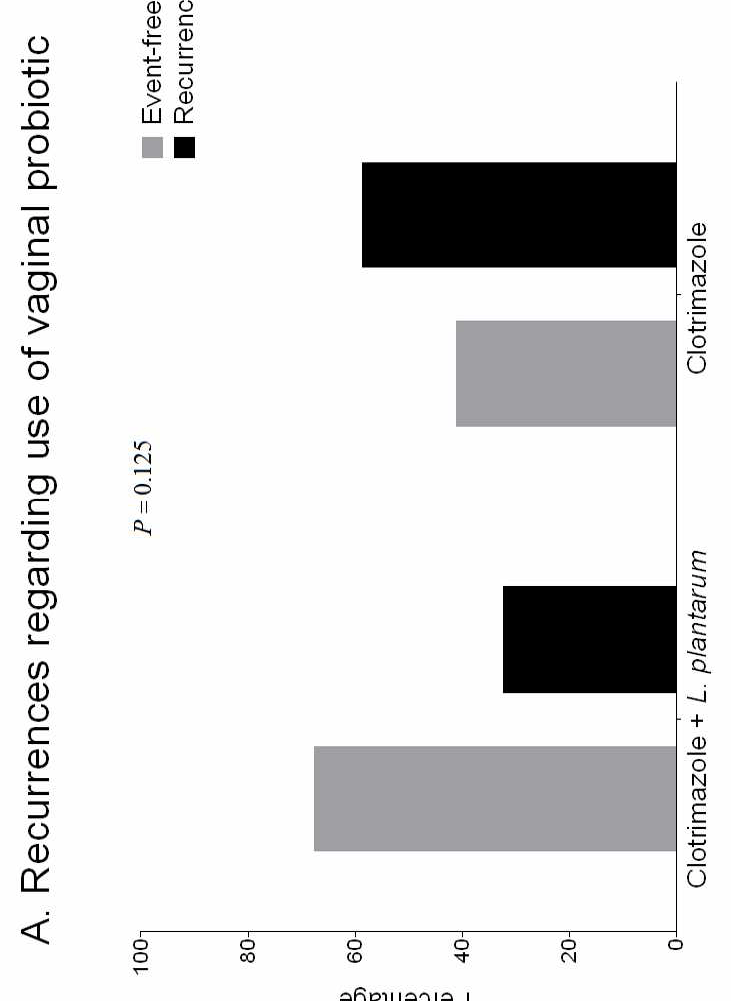

Supplement: Figure S1 — Symptomatic recurrence at 3 months according to use of vaginal probiotic (A) and antibiotic treatment prior to enrolment (B) [file 10096_2016_2715_Fig4_ESM.gif]
